# Aprepitant for refractory cutaneous T-cell lymphoma-associated pruritus: 4 cases and a review of the literature

**DOI:** 10.1186/s12885-017-3194-8

**Published:** 2017-03-16

**Authors:** Johanna S. Song, Marianne Tawa, Nicole G. Chau, Thomas S. Kupper, Nicole R. LeBoeuf

**Affiliations:** 1 Department of Dermatology, Brigham and Women’s Hospital, 221 Longwood Ave., Boston, MA 02115 USA; 2 Center for Cutaneous Oncology, Dana-Farber Cancer Institute, 450 Brookline Ave., Boston, MA 02215 USA; 3 Center for Head and Neck Cancer, Dana-Farber Cancer Institute, 450 Brookline Ave., Boston, MA 02215 USA; 4 Harvard Medical School, 25 Shattuck St., Boston, MA 02115 USA

**Keywords:** Cutaneous T-cell lymphoma, Aprepitant, Emend, Pruritus, Itch, Case report

## Abstract

**Background:**

Aprepitant is an FDA-approved medication for chemotherapy-induced nausea and vomiting. It blocks substance P binding to neurokinin-1; substance P has been implicated in itch pathways both as a local and global mediator.

**Case presentations:**

We report a series of four patients, diagnosed with cutaneous T-cell lymphoma, who experienced full body pruritus recalcitrant to standard therapies. All patients experienced rapid symptom improvement (within days) following aprepitant treatment.

**Conclusion:**

Aprepitant has been shown in small studies to be efficacious for treating chronic and malignancy-associated pruritus. Prior studies have shown no change in clinical efficacy of chemotherapeutics with concurrent aprepitant administration. These cases further demonstrate that aprepitant can be considered as a therapeutic option in malignancy-associated pruritus and further support the need for larger clinical trials.

## Background

Aprepitant (Emend; Merck & Co Inc) has been approved for use as an antiemetic in patients receiving chemotherapy. It blocks the binding of substance P to its receptor, neurokinin-1, which plays a role in pathways that induce nausea and vomiting. Recently in the literature, there have been multiple successful case reports of aprepitant use for pruritus. We report four cases of successful use of aprepitant for generalized pruritus in patients diagnosed with cutaneous T-cell lymphoma (CTCL) and review the available clinical literature.

## Case presentation

A 51-year-old woman presented with a 1.5-year history of lymphomatoid papulosis and extensive cutaneous anaplastic large cell lymphoma. The patient had experienced severe full-body itch with the diagnosis of her disease, which was moderately responsive to prednisone. She had previously been treated for her lymphoma with methotrexate and NB-UVB with no improvement in disease or itch. PUVA was tried and discontinued because of bullae development. She subsequently completed eight cycles of brentuximab, but had disease progression off treatment. She was then started on a clinical trial with an inhibitor of program death receptor 1 (PD-1), but was taken off the trial due to progressive disease. Itch persisted throughout her disease course. She began treatment with single-agent gemcitabine 6 weeks prior to the initiation of aprepitant and had persistent itch and disease with this.

On exam, she had multiple erythematous and skin-colored papules and plaques on her face, upper extremities, trunk and neck. She had no lymphadenopathy. Laboratory findings including serum chemistries, blood urea nitrogen, complete blood cell count, thyroid and liver function were normal.

Treatment with oral aprepitant, day 1, 125 mg; day 2, 80 mg; day 3, 80 mg was initiated with cycle 3, day 1 of gemcitabine chemotherapy (administered days 1, 8 of a 28 day cycle). Her symptoms improved three hours after aprepitant treatment from 10/10 to 0/10 for five days, but then her pruritus returned at 4/10 and increased thereafter until she took her next aprepitant dose with chemotherapy. On weeks where she did not take aprepitant, days 16–28, she experienced severe pruritus. She completed three cycles of gemcitabine with minimal response in her disease. Three weeks after aprepitant initiation, she underwent electron beam radiation therapy and began romidepsin. Aprepitant dosing was adjusted to every other day, with pruritus reaching 5/10 before the next dose and pruritus relief to 0/10 following every dose. Three months after aprepitant initiation, due to increased disease burden, brentuximab chemotherapy and surface conformal brachytherapy were initiated. Aprepitant dosing was then adjusted to every three days due to attempt to prolong reduced itch periods, as insurance coverage was challenging; she continued with pruritus reduction ranging from 4/10 to 0/10 for 1 year using this regimen.

Three additional patients with cutaneous T-cell lymphoma (CTCL) were treated with aprepitant for pruritus. The clinical findings of these patients are shown in Table 1.

**Table 1:** Clinical characteristics of patients with symptoms of itch treated with aprepitant therapy

Source (year)	No. of total patients	No. of responding patients	Complete vs. partial response	Itch Scale	Response by Scale	Age, y/sex	Primary cause of itch	Dose of aprepitant	Length of treatment
Patients with Malignancy Associated Itch									
Duval et al. (2009) [20]	3	3	Partial	VAS	9 to 2	N/A	CTCL, SS	80 mg/d	7d
					7 to 2				
					8 to 3				
Vincenzi et al. (2010) [21]	2	2	Complete	VAS	8 to 0	44F	NSCLC on erlotinib	d1: 125 mg; d2, 3: 80 mg; then 125 mg, 80 mg alternating	2m
			Partial		9 to 1	74 M			
Vincenzi et al. (2010) [22]	2	2	Complete	VAS	8 to 0	N/A, M	Metastatic soft tissue sarcoma	d1: 125 mg; d2, 3: 80 mg	3d
			Partial		9 to 1	N/A, F	Metastatic breast carcinoma		
Booken et al. (2011) [15]	5	4	Partial	VAS	Mean 9.8 to 4.3	56F	CTCL, SS	d1: 125 mg; d2, 3: 80 mg; every 2 weeks	Median 15w (range 6–24)
						65F	CTCL, SS		
						65 M	CTCL, SS		
						51 M	CTCL, MF		
Mir et al. (2011) [23]	1	1	Partial	Subjective	Pruritus regressed	54, N/A	NSCLC on erlotinib	80 mg/d	14d
Ladizinski et al. (2012) [24]	1	1	Partial	VAS	10 to 1	66 M	CTCL, MF	80 mg/d; 3×/week	4m
Santini et al. (2012) [14]	24	41	Complete	VAS	Median, 8 to 0	42-76 M/F	Refractory itch, metastatic solid tumor	d1: 125 mg; d3, d5: 80 mg	1w
	21		Partial		Median, 8 to 1	45-70 M/F	Naïve to treatment, metastatic solid tumor		
Torres et al. (2012) [25]	2	2	Partial	VAS	8 to 2	N/A	CTCL, SS	80 mg/d	15d, then every other d for 10d
					9 to 3				
Jimenez Gallo et al. (2013) [26]	1	1	Partial	VAS	10 to 2	41F	CTCL, MF	d1: 125 mg; d2, 3: 80 mg; every 2 weeks	N/A
Borja-Consigliere et al. (2014) [27]	1	1	Partial	VAS	10 to 3	61F	CTCL	d1: 125 mg; d2, 3: 80 mg; every 2 weeks	13m
Villafranca et al. (2014) [28]	1	1	Partial	VAS	9 to 5 after two weeks, then to 4 after one month	27F	Hodgkin’s lymphoma	80 mg/d	1m
Present cases (2017)	4	4	Complete	NRS	10 to 0	51F	CTCL, lymphomatoid papulosis/cutaneous anaplastic lymphoma	d1: 125 mg; d2, d3: 80 mg	3w; then every 3d for 12m
			Partial		10 to 6; after 8 months to 2; during non-treatment weeks pruritus increases to 6	68F	CTCL, MF		every 2w for 10m
					10 to 2; during non-treatment weeks pruritus increased back to 10	64 M			every 2w for 6m
					10 to 4	59 M			1m
Patients with Chronic Itch, Non-malignancy Associated									
Ally et al. (2013) [29]	1	1	Partial	Subjective	Vast improvement	61F	Brachioradial pruritus	80 mg/d	2w
			Complete		8 to 0	66F	Multifactorial (hyperuricemia, iron deficiency)		
					8 to 0	50F	Unknown		
Stander et al. (2010) [13]	20	16	Partial	VAS	8 to 1	42 M	Multifactorial (thyroid dysfunction, neurogenic)	80 mg/d	6.6d (range 3–13)
					10 to 3	59F	Multifactorial (metabolic syndrome)		
					10 to 4	73F	Multifactorial (renal, diabetes)		
					10 to 5	55F	Multifactorial (cholestatic, dry skin, psychosomatic factors)		
					10 to 5	52F	Unknown		
					8 to 4	78 M	Renal		
					6 to 3	72 M	Unknown		
					8 to 5	36 M	Unknown		
					7 to 4	72 M	Renal		
					5 to 3	66F	Multifactorial (renal, dry skin)		
					7 to 5	69 M	Renal		
					10 to 8	82F	Multifactorial (renal, hyperuricemia)		
					7 to 6	81F	Unknown		
					10 to 9	85F	Unknown		

*Abbreviations*: *N/A* not available, *d* days, *w* weeks, *m* months, *y* years, *VAS* visual analogue scale, *NRS* numeric rating scale, *CTCL* cutaneous T-cell lymphoma, *SS* Sezary syndrome, *MF* mycosis fungoides, *NSCLC* non-small cell lung cancer

## Discussion

Itch in the oncology patient presents an additional challenge that may dramatically affect quality of life in those already facing a cancer diagnosis and adverse effects from antineoplastic therapies. Pruritus is thought to be a multifactorial symptom that may be induced by local skin immune responses as well as global neurological pathways. Local cutaneous pathways are mediated by itch-selective C nerve fibers, whose signals are augmented by local T cells, mast cells, cytokines and neuropeptides. The C nerve fibers synapse with second-order projections, which continue to transmit signals to the thalamus for processing [1].

Aprepitant, approved for use in chemotherapy-induced nausea and vomiting in 2003, has been used with increasing frequency for this indication both as a stand-alone treatment and as part of combination regimens. This medication is well tolerated. In a systematic review including 8740 patients treated with aprepitant, statistically significant differences in fatigue and hiccups as well as infections were seen; of note the patients contributing to increased infections were from a single study where high doses of dexamethasone were used concomitantly [2, 3]. Aprepitant is a neurokinin-1 (NK_1_) receptor antagonist that can cross the blood-brain barrier; it prevents substance P from binding to its NK_1_ receptor. Substance P, a tachykinin neuropeptide, mediates nausea pathways in the brainstem as well as itch pathways from the skin to spinal cord [4]. Injected substance P into the skin of non-atopic patients induces an itch response in normal and inflamed skin [5]. Atopic dermatitis patients have been observed to have increased substance P-positive and NK_1_ receptor-immunoreactive nerve fibers as compared to healthy controls [6]. Substance P has been shown to bind NK_1_ receptors on keratinocytes, which activate mast cell degranulation and release of cytokines and chemokines such as histamine, prostaglandin D2 and leukotriene B4, which mediate itch [7]. NK_1_ receptors are also present in rat dorsal horn neurons, which may play a role in neurologic itch [8]. The importance of these neurotransmitters specifically in oncology patients has not been studied and their roles require further research.

Pruritus is sometimes a non-specific presenting complaint of underlying malignancy. While this is most often described with Hodgkin’s disease, it is also reported with many solid tumors such as those originating in the breast, gastrointestinal system and liver. In small studies of patients with non-specific generalized itching, underlying malignancy was found to be the cause of itch in fewer than 10% of patients [9]. Appropriate assessment of true, diffuse pruritus symptoms includes an age and symptom appropriate malignancy evaluation. The pathophysiology of malignancy and itch has yet to be clearly elucidated; however, many mediators have been suggested to play a role. Recent studies propose that the T-cell dysregulation associated with Hodgkin’s lymphoma contributes to high rates of pruritus associated with this malignancy [10] and the cytokines IL-6, IL-8, and IL-31 may also play roles in lymphoma-associated or chronic itch [9]. In our reported cases, patients suffered from cutaneous lymphoma, which unlike pruritus without a rash, has multiple potential contributors to itch symptoms. These patients were concurrently treated for their primary malignancy with variable response. Although malignancy treatment may also relieve pruritus, in all of our cases, patients had previously failed conventional treatments for itch for many months prior. Our cases also reported pruritus cessation within hours to days after aprepitant treatment, in the setting of progressive or persistent malignancy, suggesting that aprepitant has a direct effect on symptom relief. Prior reports also suggest that patients suffered rebound itch upon cessation of aprepitant with continuation of chemotherapy, suggesting a role for aprepitant’s direct involvement in pruritus relief.

Aprepitant is metabolized through the cytochrome P450 system, specifically CYP3A4. It moderately inhibits CYP3A4, induces CYP2C9 and possibly affects other isoenzymes. As such, medication interactions, specifically with chemotherapeutic drugs metabolized by these enzymes should always be considered [11]. However, studies in patients concurrently treated with aprepitant and docetaxel, vinorelbine or cyclophosphamide have not shown clinically significant decreases in chemotherapeutic serum concentrations [12].

To date, at least 74 patients in the literature have been reported to experience pruritus relief with aprepitant treatment (Table 1). In prospective studies, pruritus relief has been reported in 80–91% of patients, suggesting that aprepitant may be uniquely effective against itch, especially in patients with symptoms refractory to standard treatments [13–15]. Because itch pathways have not been fully elucidated and are likely activated through more than one process in the oncology patient with inflammatory skin lesions, deactivating itch likely requires a multi-faceted approach. With persistence of an inflammatory malignancy, the trigger of the itch response persists and it is expected that the itch will recur. In addition, chemotherapeutic regimens may result in modified pathways via effects on the skin and small nerve fibers. When multiple potential causes of itch exist, combination therapy with conventional anti-itch agents may be helpful; emollients, topical steroids, antihistamines, gabapentin, pregabalin, mirtazapine, ultraviolet light and tricyclic antidepressants should be considered depending on patient findings, comorbidities and with consideration of medication interactions. These can be adjusted to bridge non-aprepitant days and in instances of treatment delays due to medication access.

In small studies, aprepitant has been shown to be both safe and effective in treatment of malignancy-associated and refractory chronic pruritus. In 10 patients with an atopic diathesis, aprepitant reduced itch >40% in 9/10 patients [13]. Further research is needed to evaluate the patient population most likely to respond to aprepitant for pruritus, as it may be a tool for malignancy-associated itch, as well as in inflammatory conditions associated with chronic pruritus. However, as demonstrated in our table, there is significant heterogeneity in dosing regimens and duration of treatment. Practitioners have prescribed dosing either as 80 mg daily or in a tri-fold pack of 125 mg/80 mg/80 mg for 3 days to 24 weeks. While further studies are needed to determine the most effective dosing regimen in oncology patients, of the previous reports of six patients treated with aprepitant 80 mg daily, none experienced a complete response in pruritus. In our patients, we use tri-fold dosing of 125 mg/80 mg/80 mg, administered weekly if itch recurs after the first 3 doses. Given that our patients experienced an increase in pruritus symptoms on days without aprepitant treatment, until larger dosing studies are available, we continue treatment while patients are experiencing relief, as cessation may cause itch to return to baseline levels.

These cases demonstrate that the use of aprepitant may be helpful in patients with CTCL who experience pruritus refractory to conventional treatments. An estimated 66–88% of CTCL patients report experiencing pruritus with effects on quality of life [16, 17]. Reports that included Dermatology Life Quality Index (DLQI) score along with the visual analogue scale (VAS) demonstrated a high correlation between the two measures. Patients who experienced large improvements in pruritus symptoms by VAS had similar dramatic decreases in DLQI scores. The VAS is a commonly used tool for quantifying itch. It has been validated in patients with chronic itch or pruritic dermatoses with a high correlation with the numeric rating scale [18, 19]. Future studies exploring how itch impacts patient quality of life and the effectiveness of interventions such as aprepitant should consider patient-reported outcomes. A disease-specific scale may be of significant use in the oncology population.

## Conclusion

We observed 4 cases of significant pruritus improvement in CTCL patients treated with aprepitant, supporting that this modality can be useful in treating some patients with malignancy-associated itch refractory to conventional treatments. There continues to be a need for larger comparative effectiveness trials of aprepitant in patients with chronic, malignancy and treatment-associated itch. Review of the literature demonstrated discrepancies in dose and timing of aprepitant regimens as well as outcome measures; prospective studies should evaluate dosing to discern the most effective administration schedule. Future study is imperative as oncology patients may experience negative impact on quality of life from treatment refractory pruritus.

## Abbreviations

CTCL: Cutaneous T-cell lymphoma
DLQI: Dermatology Life Quality Index
NK_1_: Neurokinin-1
VAS: Visual analogue scale
